# Reaching Thousands of Children in Low Income Communities With High-Quality ECED Services: A Journey of Perseverance and Creativity

**DOI:** 10.3389/fpubh.2021.637031

**Published:** 2021-03-26

**Authors:** Nathalia Mesa, Milagros Nores, Hilda Vega

**Affiliations:** ^1^Fundacion Carulla-aeioTU, Bogotá, Colombia; ^2^National Institute for Early Education Research, Rutgers University, New Brunswick, NJ, United States; ^3^Independent Philanthropic Advisor, Hispanics in Philanthropy, Chicago, IL, United States

**Keywords:** early childhood development, early childhood development centers, social enterprise, public-private partnerships, Reggio Emilia, Reggio Emilia approach, scalable models

## Abstract

This paper describes a creative and bold way in which a local NGO addressed increasing access and quality of ECED services in Colombia. This case study on Fundacion Carulla's aeioTU early childhood innovation in Colombia contributes to understanding the possibilities for the private sector to spark innovation, and the importance of an open and collaborative strategy in contributing to the ECED sector at large. The critical role of monitoring and evaluation in the provision of services is highlighted. This guided key decisions on different growth phases. After a decade of work, Fundacion Carulla-aeioTU has shown capacity to effectively support children's development in low-income settings through their participation in quality programming. Furthermore, this case study also describes how the organization, having proven its capacity to provide high-quality services directly to children, decided to innovate and bring about different solutions to reach and support other stakeholders in the early childhood development ecosystem.

## Introduction

For decades, the infrastructure of early childhood education and development (ECED) services for low-income children in Colombia had been inadequate and insufficient, mirroring profound social and economic inequities across the country. In 2008, after research and consultations with various institutions including small ECED centers and government agencies, Fundación Carulla (FC) decided to create a new social enterprise—called aeioTU—to provide high-quality ECED services to children through direct services as well as a social franchise model. In addition, FC identified a need to develop the early childhood education and development cluster across the country, in order to expand and strengthen high-quality ECED services for low-income children.

The original aeioTU business plan included the creation of 19 ECED centers in high-income communities that—through their profits—would subsidize 4 aeioTU centers in low-income communities. It also included 65 self-sustaining aeioTU social franchise centers. Ultimately, this would reach an estimated 33,608 children and encompass 1,364 teachers.

During the first 5 years of implementation, aeioTU defined its operational model and piloted its franchise-based business model, driving for sustainability, quality inclusive services and evaluating the program's impacts. After a decade of building programs, adjusting programming and priorities and investing in building a cluster for ECED advocacy, aeioTU has reached 228,667 children in 1,851 ECED centers working with 17,238 teachers. aeioTU is on its way to becoming and impactful, sustainable social enterprise reaching programs and families in Colombia, other parts of Latin America and even Africa.

This paper provides a detailed description of how this ECED program was built and scaled-up in Colombia, and of how it engaged with a variety of national and international stakeholders to promote country-level change in ECED services. The paper illustrates the various stakeholders that can be engaged in ECED, as well as highlight alternative long-term strategies for sustaining ECED growth in developing contexts. It describes the relationship between a private foundation and local and national governments; the adaptation of quality programs to local contexts, and the use of measurement and evaluation for improvement, adaptation and scaling.

## The Landscape of Early Childhood Education in Colombia

In 2008, the critical importance of investing in ECED had gained ground globally and across diverse sectors in Colombia (1). The national government, a few large city governments, NGOs and universities had been working on advancing early childhood development, but significant gains had not been made (1). There were substantial gaps in the quantity and quality of ECED services across the country. Of an estimated 4 million children in the country, 35% experienced multidimensional poverty with only 33% having access to some type of ECED program (2, 3). There were no national policies on early childhood programming or services. It was not until 2006 that early childhood education was established as a fundamental right (4).

The national government was funding two large programs; one that provided children with a breakfast and the *Hogares Comunitarios* program, a home-based care program (5). The latter was the only ECED program evaluated in the country and showed weak impacts. According to the National Planning Department's (DNP) evaluation of the program in 2009 (6), despite positive results in terms of hygiene conditions and psychosocial development, there was no evidence of cognitive impacts on children and some indication of negative impacts on health (6).

A broad overview of the ECED sector in Colombia carried out by FC identified a highly fractured sector with low demand for early childhood services and low capacity to pay for these. This overview further showed services were relatively small-scale, evidenced little innovation and no monitoring or evaluation. More specifically, there were no agreed-upon criteria for defining quality in early childhood programs.

Education and health sectors lacked strong vertical and horizontal integration (1), despite a history of integration of early services exemplified with the creation of the Instituto Colombiano de Bienestar Familiar (ICBF) in the 1970s (1). Funding for services was also unstable (1). The conditions for adequate provision of services were weak, with centers and classrooms lacking proper infrastructure, didactic materials, and early childhood qualifications for teachers, and with an absence of standards, monitoring and evaluation. Despite the evidence on the importance of qualified and trained teachers in early education, there was no emphasis on qualifications or professional development of early childhood teachers at the time. According to a 2015 UNESCO report Colombia was not producing enough professionals to meet the national ECED strategy (*De Cero a Siempre*), with only 7,500 professionals graduating from relevant programs annually and 74,000 needed (7).

In ECED programs, common practice was to hire teachers on short-term (10–11 months) contracts, contributing to job insecurity and high turnover. Some cities did not have professional development budgets (8). The *Hogares Comunitarios* program caring for young children paid less than the minimum salary to its estimated 800,000 community mothers, each caring for 10–12 multi-age children in their own homes (8).

In 2008, Colombia was the second largest country by population in South America (42.25 million), with a diverse geography and distinct regional cultures, demanding innovation in infrastructure and capacity to reach remote communities. The rich and varied cultural heritage can be supportive of children's educational experiences, but programming at scale was challenging given the differences between urban and rural contexts and the varying levels of human capital across the regions. Despite being the longest standing democracy in the region, with decentralized governments and a tradition of free enterprise and market solutions, the country continued to experience internal violence and migration (1). A growing migration from neighboring Venezuela was becoming tangible.

In this landscape, FC met with the National Secretary of Education of Colombia in October 2007 to understand in which education sector FC could have the greatest impact. The Secretary recommended supporting the development of a national ECED strategy that would build on the growing evidence on the importance of early interventions for children's development and school performance. There was yet no national ECED policy. The board of FC decided to commit its efforts to promoting system-wide change for children ages zero through five through investment in innovative, bold, high-quality and sustainable programming. This plan would include direct provision, as well as public and private partnerships.

## A Social Entrepreneurship Approach to Early Childhood Education

FC developed a business plan to create “model” ECED centers under the brand aeioTU. These would then serve as prototypes for *other* providers to replicate as franchises. Franchises would benefit from access to aeioTU's start-up financing, facilitated monitoring of early child development, and linkages to professional development and cross-center collaboration. The idea was that model centers and franchises would generate economies of scale in negotiations with suppliers, and most importantly, stimulate system-wide change. FC sought to become a driving ECED actor contributing to high impact, sustainable change through its vision and a strong business model. FC was guided by the following learning questions:

- Can we create a high-quality ECED model center at low cost despite the challenging context in Colombia?
- Can we scale the ECED model center to reach thousands of children?
- Can our example lead a transformation of the ECED sector in Colombia, mobilizing providers to advance high-quality ECED services to low-income children across the country?

## Inspiration on the Reggio Emilia Approach

Of the many ECED experiences around the world and within Colombia, FC was most inspired by the Reggio Emilia approach (9). FC had a vision of contributing to the transformation of Colombia through the development of and social commitment to its children, to move the country toward greater levels of child development, social mobility, environmental awareness, democratic values, social inclusion, peace and innovation. The history of the Reggio Emilia approach resonated with FC because it originated in Italy as it transitioned from a period of violence after World War II. Reggio Emilia's values of hope in the ability to rebuild a community; collaboration with local families and communities; creation of social capital; empowerment of children, educators and families; recognition of and value in one's identity, and viewing children as the drivers of the educational experience were all critical to shaping FC's own vision. More so considering the context in Colombia, which at the time continued to experience internal violence and related internal migration (10).

## Programmatic Elements

FC launched aeioTU with the understanding that the relationships and interactions children had with themselves and with adults, and the relationships among adults in the ECED centers, were key to high-quality ECED services. The aeioTU centers, would promote high quality by focusing on 6 critical areas: a comprehensive combination of nutrition, health and education objectives, clear pedagogical objectives and a curriculum with an emphasis on continuity across the early years, continuous professional development, adequate physical space and materials, family participation, transition to formal schooling, strong center management and planning for sustainability (9). While aeioTU predated the release of the nurturing care framework (11), it similarly takes a life-course perspective that encompassed good health, adequate nutrition, responsive caregiving, and opportunities for early learning. The nutritional component includes child nutritional monitoring and providing 70% of their nutritional intake needs. The program also includes engaging parents on nurturing care and positive discipline. aeioTU is an inclusive program and has strategies around inclusion that include family engagement, teacher training, parent training and developmental follow-ups (this component has not been evaluated independently).A comprehensive description of the model is included in Nores et al. (9).

aeioTU's goal was to scale and reach as many children as possible to provide a strong start for children across all developmental domains, including socio-emotional development. The initial business plan approved the creation of 19 for profit aeioTU centers, 65 sustainable centers where families contributed to operational costs and 4 fully subsidized centers in low-income communities. The latter where to be funded from outside sources and the subsidizing for-profit centers. This social enterprise model was to generate high-quality ECED services for 33,608 children in a period of 10 years, via 84 centers employing 1,364 teachers. Home-visitation components were developed later under *aeioTU en casa* (aeioTU at home).

## Direct Operation of aeioTU ECED Centers

In its first year, the aeioTU service center was created with a matrix organization that included pedagogy, partnerships, finance, administration and communications teams to support the start-up and operation of three new aeioTU centers. This first phase included the development of standards, curriculum and guidelines for the educational experience.

In January 2009, aeioTU opened its first for-profit center in Bogotá, and two fully subsidized centers in low-income communities in Barranquilla and Bogotá. Each center included a center coordinator, a team of teachers, a psychologist, an artist, a nutritionist, an administrator and kitchen staff. The centers' teams focused on engagement with the local community and context—key social actors, families, and the environment—, and the process toward opening the centers included community consultations. Teacher to student ratios (at the beginning) were 1:12 with at least 3 m^2^ of physical space per child in the classroom. The centers served children birth through age five, with class sizes increasing by age.

By 2011, the centers had been well-received by the local communities and the ECED sector at large. Centers were at capacity or had waiting lists. With media coverage, recognition for the model grew. City governments, companies, and grant-making foundations started to show interest in the model. The President of Colombia and the ministries of education, health, and social services visited the centers and remarked on the innovations in pedagogy, staffing, and materials. President Alvaro Uribe visited the first aeioTU center on 2009 when the center was officially inaugurated (12), and President Juan Manuel Santos held its nation-wide planning meeting on ECED in an aeioTU center in Santa Marta on 2011 (13).

**Figure 1:**
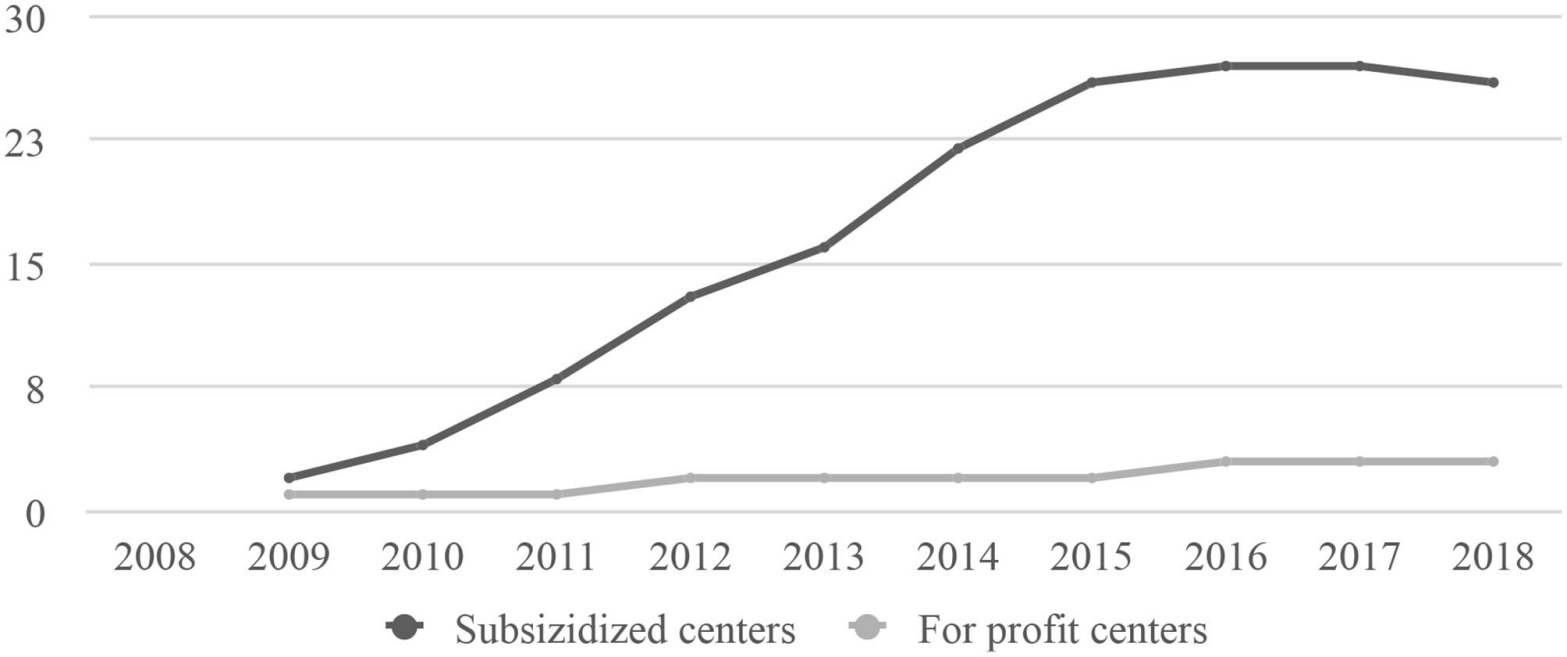
Growth of aeioTU centers by type 2009–2019.

After opening the initial centers, the initiative started to evolve to focus on continuous improvement of the learning experience, standardization, quality certification, and scalability. A longitudinal randomized controlled study was initiated in two of the newer centers (10). The growth of aeioTU centers over the next decade by funding type is portrayed in Figure 1.

aeioTU opened an average of 3 centers per year from 2008 through 2016 in 15 cities. These included large urban environments, with palpable urban violence, as well as medium-sized cities and small rural communities. The growth was motivated by the idea of learning and piloting the aeioTU ECED learning experience in a variety of communities, as well as by the demand for the aeioTU model emerging from communities, companies, foundations and local governments. This growth of subsidized centers deviated from the original business plan, but was funded by tens of organizations willing to commit to this national experiment, which aeioTU engaged with as partners. Three key areas of the operation were evaluated and improved upon during this 8-year period of growth, along with efforts to optimize the per-child cost and improve the quality of the learning experience:

- ***The for profit aeioTU centers:***a first center opened as planned, but it took some time to reach a point when no subsidies were needed. This delayed the opening of additional for-profit centers. Two of these centers exist today. As described by a mother of this first center “It has been marvelous for us as a family to discover the social work that the Foundation does by providing the same educational model to low-income families. We are aware of how fortunate we are to have the means to provide our girls with the best education; and are happy to participate somehow in providing the same model to children with less opportunities. That is precisely the principle of solidarity that we want to teach our children. Like aeioTU, we know education is key to reduce the inequalities that exist in our society” (14).
- ***The sustainable aeioTU franchise:***franchises were never initiated because the cost of operating the centers was not low enough for sustainability in this model under which middle-income families would pay tuition. The aeioTU “company center” was instead created, with companies financially supporting a center for its employees' children. Two of these centers exist to date.
- ***The subsidized centers:***Direct provision of ECED services in low-income communities had such a strong response and faced such high demand that aeioTU grew significantly more than originally planned. Most of the funding came from the public sector and philanthropy, rather than from the for-profit centers described above. For-profit centers have however funded 577 children. The government and philanthropy have funded 22,994 children.

In low-income communities, where demand for slots was significant, centers of 300 children proved to be optimal in terms of per-child cost (larger than typical centers of 60–100 children in Colombia).

In order to reach lower the per-child cost while preserving quality, aeioTU innovated in the use of space, the weekly schedule, the type of buildings, classroom equipment and materials. This also included shifting in some locations to *aeioTU en casa* (aeioTU at home) to support pregnant moms, infants and toddlers. Nutritional services were eventually shifted to a specialized organization that worked in partnership with the centers, to ensure reasonable pricing and quality at a larger scale. This allowed leadership teams to focus on the pedagogical components. Appendix 1 displays the innovations and changes made throughout the decade toward sustainability.

A central component of aeioTU has been an emphasis on evaluation and continuous quality improvement. Between 2008 and 2013 aeioTU underwent two external evaluations that drove improvements: (a) a longitudinal study conducted by Rutgers University and Universidad de los Andes (9, 15), and (b) visits from Reggio Children. In particular, the longitudinal study evidenced positive cognitive and health impacts (as measured by anthropometric indicators) on children early on (9) and sustained medium-term (Bernal et al., unpublished). Programmatic improvements were a direct response to lessons from these evaluations including improvements in:

- understanding and modifying the day schedule for children to reduce time spent in transitions and strengthen the education component,
- materials and use of space,
- the use of documentation and research by teachers,
- the role of the teachers within the classroom,
- the relationship with the community, including the social and physical environment,
- the creation of a system of quarterly indicators to follow children's development, used in meetings with the family,
- the development of ConecTU, a tool to systematize and generate reports with aggregated child information,
- the strengthening of professional development (PD) for aeioTU teachers and families, and PD supports,
- shifting services for babies and toddlers to integrate services starting at birth.

Claudia Giudicci, Reggio Children president, wrote in 2016 to aeioTU “I [renew] my gratitude and that of Reggio Children for the extraordinary work that you are doing in Colombia with aeioTU to provide children with a new future….your efforts promoting the rights of children is notable” (16). Ellen Frede, as co-director of NIEER wrote “I was fortunate to visit the centers, meet the teachers, center coordinators, parents and educational leaders to revise and comment on the materials and procedures that are included in the highly complex but manageable system of the Curriculum Cartography. This system and materials are a great contribution to early learning… it is a resource to the world” (17).

The challenges of working in regions with local conflict proved to be unique. aeioTU innovated using art, partnering with the family and the community in order to preserve the centers as safe and peaceful spaces within the neighborhoods, while eliminating the use of security guards. There has never been violence inside the centers, and the families themselves returned the few times items robbed from a center. Having independent and empowered female teachers created some discomfort in one community, and was ascribed to a patriarchal system. Consequently, aeioTU included diagnostic tools and PD to support teachers in engaging with such complex situations.

Since its creation, aeioTU used the Balanced Scorecard system (BSC) to manage its work. The BSC included a strategic map with objectives, indicators and annual initiatives to achieve intended goals. The management team reviews the results of indicators and key initiatives monthly, the Board does so quarterly, and these are revised yearly. Appendix 2 has a copy of the strategic map used and Appendix 3 includes a copy of the theory of change under which the BSC operates.

It is important to note that the decentralized government model of Colombia has meant that aeioTU has had to work with multiple government stakeholders including the national government and 13 provincial governments. Yet, due to free enterprise policies in Colombia, aeioTU was able to create the cross-subsidy model, operate aeioTU centers with public and private funding, and leverage multiple sources of funding across all its centers, including resources from other NGOs.

According to Maria Clemencia Rodriguez de Santos, former first lady of Colombia from 2010 to 2018, “The [*Cero a Siempre*] policy, established by the administration of President Juan Manuel Santos and from which I was the spokesperson, was made possible by the joint commitment of the children's families, the public and the private sector, who allowed the comprehensive care for early childhood to become a reality. In this endeavor, aeioTU was a committed and unconditional ally” (18).

## Scaling the aeioTU Model for Greatest Reach and Impact

By December 2015 aeioTU had 28 ISO 9001 certified centers for 13,315 children, with stability of contracts and staff and showed high satisfaction of employees and partners. The centers existed in different contexts and size configurations. Therefore, with the support of the Interamerican Development Bank, in late 2015 aeioTU worked with a social franchise expert from London to prepare the social franchise business plan. The 2015–2025 business plan envisioned 20 aeioTU franchises and ten new directly operated centers. At the Board Meeting in December 2015, the recommendation was to go back to the drawing board because the social franchise model was too expensive and difficult to implement in terms of quality and consistency for space, materials, and PD. It would require costly monitoring and supervision.

The management team continued to research scaling alternatives and in 2016 piloted two efforts to engage partners in replicating impact, to reach many more children indirectly.

- A collaboration with the National Government to work with 300 ECED centers serving an estimated 45,000 children in low-income communities in the northern coast of Colombia. The goal was to improve these centers' quality and processes. The program was evaluated by Universidad de los Andes (19).
- A partnership with Corpoayapel, an NGO in the province of Cordoba, Colombia, serving 6,000 children. aeioTU shared knowledge and provided support. The LEGO Foundation funded an evaluation of this endeavor, which showed positive results (20).

By December 2016, aeioTU had gained worldwide recognition as an innovative solution for ECED. Nathalia Mesa, CEO of aeioTU since its initiation, was selected as an ASHOKA Fellow. AeioTU participated in the Ashoka Globalizer program, under the ReImagine Learning Initiative of the LEGO Foundation (21). This allowed aeioTU to embark on 6 months of strategic planning, and decided to shift to a flexible strategy that included three solutions for expanding beyond the directly operated centers:

1. AeioTU *Aprendiendo* (22), an internet platform where documents, videos and PD are provided for free, accompanied by short term additional PD at low cost. This platform has been key with the move to remote instruction during the COVID-19 pandemic.
2. One-year PD modules for centers to support quality improvements.
3. The aeioTU Network membership, which includes information-sharing, networking and fundraising for partners operating across the country.

This new business model preserves the intent of the original plan, but also recognizes the role of the aeioTU centers in the ECED system at-large. The social franchise model evolved from a closed, tightly controlled strategy to a more fluid knowledge-sharing strategy were other ECED centers are recognized peers in a network, and are supported in efforts toward increasing quality for a larger number of children. Ruth Gomez, who oversees an estimated USD1M investment of aeioTU supporting a low-income neighborhood of about 10,000 children in Cartagena discusses this strategy:

“For the Fundacion Grupo Social it is welcoming to work with FC-aeioTU to achieve the transformation of the early education ecosystem… its pedagogical experience validated and recognized in Colombia gives us tranquility that it will contribute to the transformations necessary to achieve an improvement in the quality of life in our territory...we have seen how the community mothers, teachers and leaders have started changing their pedagogical practices and strengthening the services to children. The new materials and improvement in spaces has developed the autonomous learning of children, improving the education and strengthening the role of the family and their interaction with their community” (23).

These three new strategies were received positively by the ECED cluster in Colombia, as shown by the subsequent growth across all three. The aeioTU PD events gathered thousands of teachers. The 1-year PD modules showed very positive evaluation results (19) and continue to be provided to other centers serving low-income children across the country, and around the world. These are funded by the national government, local governments and philanthropies.

aeioTU's network reached 9,000 children in the first year—a challenge made possible with aeioTU's support in partnerships, financial planning, human resources and pedagogy. This also created economies of scale for buying materials and supplies.

Government and grant making organizations supported further expansion of the training programs, while other ECED centers embraced aeioTU practices, including PD for their teachers. ECED center directors and teachers improved their operations, thousands of teachers participated in the online learning platform, newly trained teachers tried new pedagogical strategies and methods to benefit children. Results provided by the independent evaluation included (19):

- Classroom transformation,
- Family involvement,
- Increased work of children in creative ways,
- Evolution from free play to imaginary play,
- Increased reading and books available for children,
- Introduction of exploration activities and/or projects for the local community and environment

Under the work with the Globalizer Program, aeioTU was able to identify the ECED ecosystem and its challenges. This awareness shifted its focus outwards, to the ECED system at large. It allowed aeioTU to understand there are as many ECED ecosystem in Colombia, as there are cities and neighborhoods. Being in 13 different communities aeioTU had to assess and define a strategy for each local context. In some communities, aeioTU then opted to collaborate with others in transforming ECED services. As of December 2019, aeioTU has reached 228,667 children in 1,851 ECED centers working with 17,238 teachers, including in other parts of Latin America (24) and even Africa (25).

According to Constanza Alarcón, current Viceminister of Education of Colombia, “FC-aeioTU ventured into the field of ECED within a historic chapter in which Colombia was structuring its early education policies and programs in a comprehensive care approach at a national level. Their valuable experience in the development of an initial educational curriculum, based on evidence, contributed to the country's challenge of increasing coverage of programs with quality, and since then has enriched the State's work in this field, setting up innovative experiences. Its articulation with the private sector and with the national and local government over the years has made it possible to show that comprehensive care for vulnerable children is a responsible, sustainable investment with a high impact on children, their families and communities” (26).

## Discussion and Conclusion

AeioTU has proven that it is possible to operate high-quality ECED centers in low-income communities in a variety of contexts. At its inception, the aeioTU model was expensive, so intentional efforts to lower cost were critical for scalability and sustainability. Now, aeioTU is on its way to becoming financially sustainable while having proven it is a high-quality and high-impact model across diverse communities. A key lesson is that a cross-subsidy model works well in contexts of high-income inequality if there is a small fraction of families able and willing to pay for high-quality services. In addition, central and local governments are willing to work on needed infrastructure beyond their individual political administrations, which is imperative for large-scale efforts. Moreover, the work of aeioTU in communities of violence and trauma further proves that ECED programs can be a feasible solution to working with children in such complex contexts. Finally, while the initial phases of the aeioTU experience predated the Nurturing Care Framework (NCF) more recently established by the World Health Organization (11), the model does in fact encompass various aspects proposed by the model included in NCF, and extends these beyond age 3. The ability of the program to comprehend health monitoring, provide nutrition, develop resources and supports for responsive caregiving, and provide early learning opportunities, as well as strengthen these in other providers, makes aeioTU a particularly interest case study in relation to the NCF.

Other core lessons and results after a decade of work include:

- **Adaptability of the aeioTU educational experience**. It is feasible to adapt a globally known curriculum for use in low-income communities with scarce resources.
- **Impact over internal growth**. The aeioTU team learned that growing an organization is not the same as growing its impact. An emphasis on the former is central for sector growth over the growth of a specific model.
- **Impact for both children and teachers**. Evaluations demonstrated an impact on children (15) and an impact on teachers (9).
- **Impact on the ecosystem**. The ECED ecosystem evolved from a fragmented, low-quality effort to a more integrated and higher quality, nation-wide system. This required that aeioTU evolve from thinking primarily about the families it served to opening itself to supporting other ECED initiatives.
- **Partnership building**. aeioTU learned the importance of connecting local, national and global actors promoting a high-quality ECED agenda to reinforce and increase impact.
- **Continuous learning**. aeioTU realized that a true learning organization evolves, adapts and engages with others, receiving feedback from stakeholders, data and evaluations for improvements prioritizing the collective goals and not just the organization.

Once an innovative solution is proven to work, how to scale becomes critical for impact. Finding the answer to this means continuous thought on the core components of a program and, finding alternative mechanisms to spark innovation beyond the boundaries of a program. For aeioTU, this required leadership willing to innovate and understanding the initial experiences of the program as usually the most imperfect. Only through data, commitment and innovation can it have large-scale impact. This has been at the core of aeioTU. Key components for success were understanding of the specific ecosystem, communicating a vision to key stakeholders and shifting from thinking about aeioTU to thinking systemically. Unfortunately, the social and educational sectors in many countries operate within contexts of scarce resources and individual sustainability. The incentives needed for sharing, co-constructing, and cross-sector supports are often low. System change requires stakeholders to build collaboratively and integrate resources.

Looking back at when aeioTU started, several skeptics thought a large-scale operation of Reggio Emilia inspired programs in low-income communities was impossible. A social enterprise business model proved to be an effective way to spark this innovation. However, the social enterprise model also proved too narrow. The adaptation that allowed for local ECED programs to improve in different ways—as well as the work of engaging in a large-scale learning experiment by sharing knowledge—proved more successful. The aeioTU experience proves it is possible to bring high-quality ECED services to any child anywhere. Public and private sectors can work together in transforming ECED ecosystems. A comprehensive vision and encompassing engagement of partners is key of the aeioTU model's ability to engage in large-scale systemic change. The need for encompassing partnerships is referenced in the NCF and its appeal for engagement across all relevant stakeholders. The aeioTU experience has therefore proven the NCF vision is feasible in low and middle-income countries.

## Data Availability Statement

The original contributions are presented in the study. Data availability is not applicable for this contribution. Further inquiries can be directed to the corresponding author.

## Author Contributions

All authors listed have made a substantial, direct and intellectual contribution to the work, and approved it for publication.

## Conflict of Interest

NM has had the following roles in Fundacion Carulla-aeioTU: Executive Director (2008-2016), Chairperson (2016-2018), Member of the Board (2018-2020), and President of the Board of Fundacion Carulla-aeioTU (Q1/2020), Bogotá, Colombia. The remaining authors declare that the research was conducted in the absence of any commercial or financial relationships that could be construed as a potential conflict of interest.

## References

[1] CINDE. Politica de Primera Infancia. (2021). Available online at: http://www.cinde.org.co/PDF/Politica%20publica%20primera%20infancia%20Colombia%20(v.%2011%20nov%2006).pdf (accessed March 11, 2021).

[2] DANE. National Quality of Life Survey Annex 18 Total Population and Percentage Distribution by Five-Year Age Groups, According to Regions of the Country and Area (Head and Rest). Colombia: DANE (2008). Available online at: https://www.dane.gov.co/index.php/estadisticas-por-tema/salud/calidad-de-vida-ecv/encuesta-nacional-de-calidad-de-vida-2008/anexos-calidad-de-vida-2008 (accessed March 11, 2021).

[3] Departamento Nacional de Planeación DNP. Analysis Results Poverty Multidimensional 2008-2014. Colombia: DNP (2008). Available online at: https://colaboracion.dnp.gov.co/CDT/Desarrollo%20Social/Analisis%20resultados%20Pobreza%20multidimensional%202010%20-%202014.pdf (accessed March 11, 2021).

[4] Ministerio de Educación. Antecedentes Política Educativa de Primera Infancia. Colombia: MEN (2021). Available online at: https://www.mineducacion.gov.co/primerainfancia/1739/article-177829.html (accessed March 11, 2021).

[5] Vargas-Barón E. Going to Scale: Early Childhood Development in Latin America. Washington: The RISE Institute (2009).

[6] Departamento Nacional de Planeación DNP. Evaluation impact of the Program Community Homes ICBF Welfare. Colombia: DNP (2009) Available online at: https://www.icbf.gov.co/sites/default/files/impacto_hcb.pdf (accessed March 11, 2021).

[7] UNESCO. A Review of the Literature: Early Childhood Care and Education (ECCE) Personnel in Low- and Middle-Income Countries. ED 2015. France: UNESCO (2015). Available online at: https://unesdoc.unesco.org/in/documentViewer.xhtml?v=2.1.196&id=p::usmarcdef_0000234988&file=/in/rest/annotationSVC/DownloadWatermarkedAttachment/attach_import_9bd23202-5460-46fa-8a25-f3ed33ce618d%3F_%3D234988eng.pdf&locale=es&multi=true&ark=/ark:/48223/pf0000234988/PDF/234988eng.pdf#2478_15%20Review%20Literature_ECCE_INT.indd%3A.21715%3A398~ (accessed March 11, 2021).

[8] Sentencia T-628/2012, de 10 de agosto, Corte Constitucional Programa Hogares Comunitarios de Bienestar: Colombia. Available online at: https://www.corteconstitucional.gov.co/relatoria/2012/T-628-12.htm (accessed March 11, 2021).

[9] Nores M Figueras-Daniel A Lopez MA Bernal R. Implementing aeioTU: quality improvement alongside an efficacy study-learning while growing. Ann N Y Acad Sci. (2018) 1419:201–17. 10.1111/nyas.13662 29791734

[10] Engel S Ibáñez AM. Displacement due to violence in Colombia: a household-level analysis. Econ Dev Cult Change. (2007) 55:335–65. 10.1086/508712

[11] World Health Organization. Nurturing Care for Early Childhood Development: a Framework for Helping Children Survive and Thrive to Transform Health and Human Potential. World Health Organization (2018). Available online at: https://www.who.int/maternal_child_adolescent/child/nurturing-care-framework/en/ (accessed March 11, 2021).

[12] Archive of the Presidency of Colombia. Available online at: http://historico.presidencia.gov.co/discursos/discursos2009/febrero/piesd_04022009.html (accessed February 4, 2009).

[13] Acuerdos y compromisos para la prosperidad de la primera infancia de Colombia. Available online at: http://www.deceroasiempre.gov.co/Prensa/2011/Paginas/111104.aspx (accessed November 4, 2011).

[14] Mambi T. Email Message to Nehyi Quintero, Communications Coordinator of aeioTU [Own translation] (2018).

[15] Nores M Bernal R Barnett WS. Center-based care for infants and toddlers: the aeioTU randomized trial. Econ Educ Rev. (2019) 72:30–43. 10.1016/j.econedurev.2019.05.004

[16] Claudia G. President of Reggio Children Srl, Email Message to Maria Adelaida Lopez from aeioTU (2018).

[17] Frede E. Co-director for the National Institute for Early Education Research at Rutgers University, Email Message to Maria Adelaida Lopez From aeioTU (2015).

[18] de Santos R Clemencia M. WhatsApp Message Sent to 1st Author. [Own translation] (2021).

[19] Maldonado C Escallón E. Evaluación del Programa de Fortalecimiento aeioTU. Universidad de los Andes [Informe 3] (2015).

[20] Maldonado C Escallón E Guerrero P Rozo L. Evaluación de la implementación del modelo aeioTU en Centros de Desarrollo Infantil del departamento de Córdoba. Universidad de los Andes [Informe 4] (2016).

[21] Ulloa E. El modelo educativo aeioTU es reconocido internacionalmente por sus altos estándares de calidad. El Mundo al Instante. Available online at https://elmundoalinstante.com/modelo-educativo-aeiotu-reconocido-internacionalmente-altos-estandares-calidad/

[22] Fundacion Carulla – aeioTU (2021). Available online at: https://aprendiendo.aeiotu.com/ (accessed March 11, 2021).

[23] Gomez R. Email Message Sent to Nehyi Quintero From aeioTU. [Own translation] (2018).

[24] Gobierno de Mexico. Aplicará SNDIF nuevo modelo de enseñanza en centros de atención infantil comunitarios. (2021). Available online at: https://www.gob.mx/difnacional/articulos/aplicara-sndif-nuevo-modelo-de-ensenanza-en-centros-de-atencion-infantil-comunitarios-216747?idiom=es (accessed March 11, 2021).

[25] Apolitical. From Unemployed to Entrepreneur: South Africa's New Preschool Educators. Available online at: https://apolitical.co/en/solution_article/unemployed-entrepreneur-south-africas-new-preschool-educators (accessed March 11, 2021).

[26] Alarcón C. WhatsApp Message Sent to 1st Author. [Own translation] (2021).

