# Supplementary figures and images for: Reaching Thousands of Children in Low Income Communities With High-Quality ECED Services: A Journey of Perseverance and Creativity
Source: Front Public Health. 2021 Mar 26;9:637031. doi: 10.3389/fpubh.2021.637031 (PMC8032966; doi:10.3389/fpubh.2021.637031)

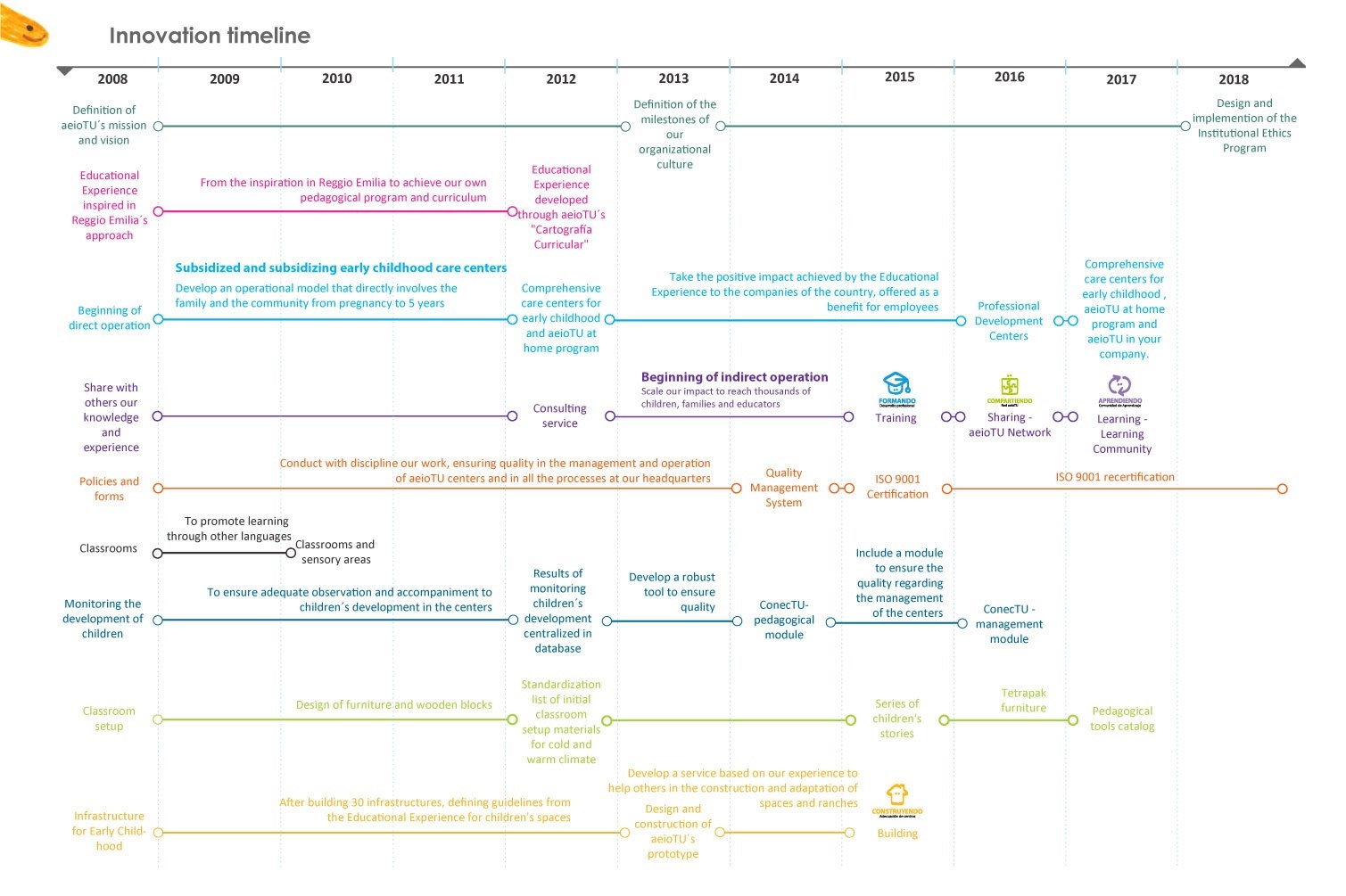

Supplement: Supplementary file 1 [file Image_1.jpg]

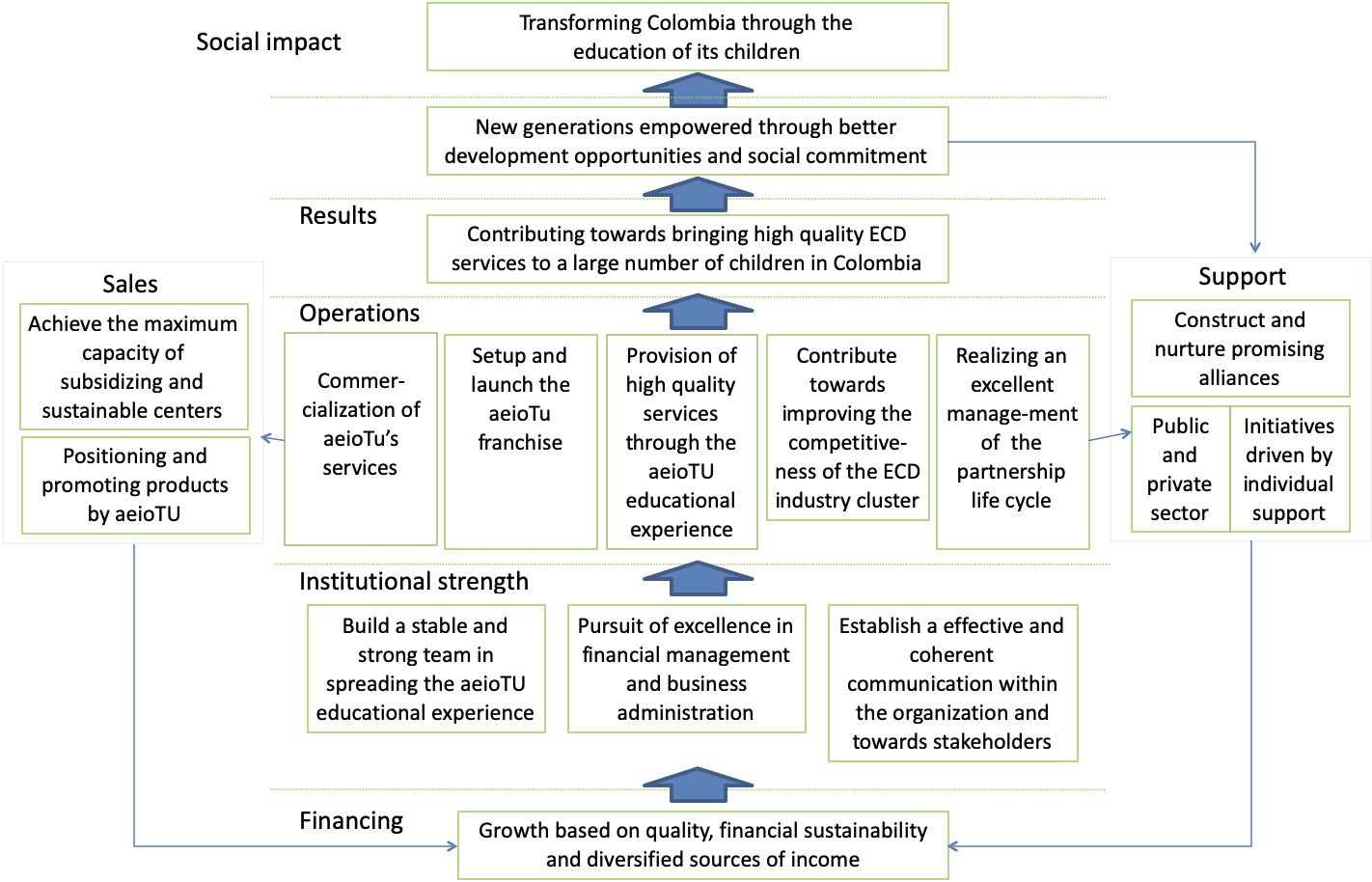

Supplement: Supplementary file 2 [file Image_2.png]

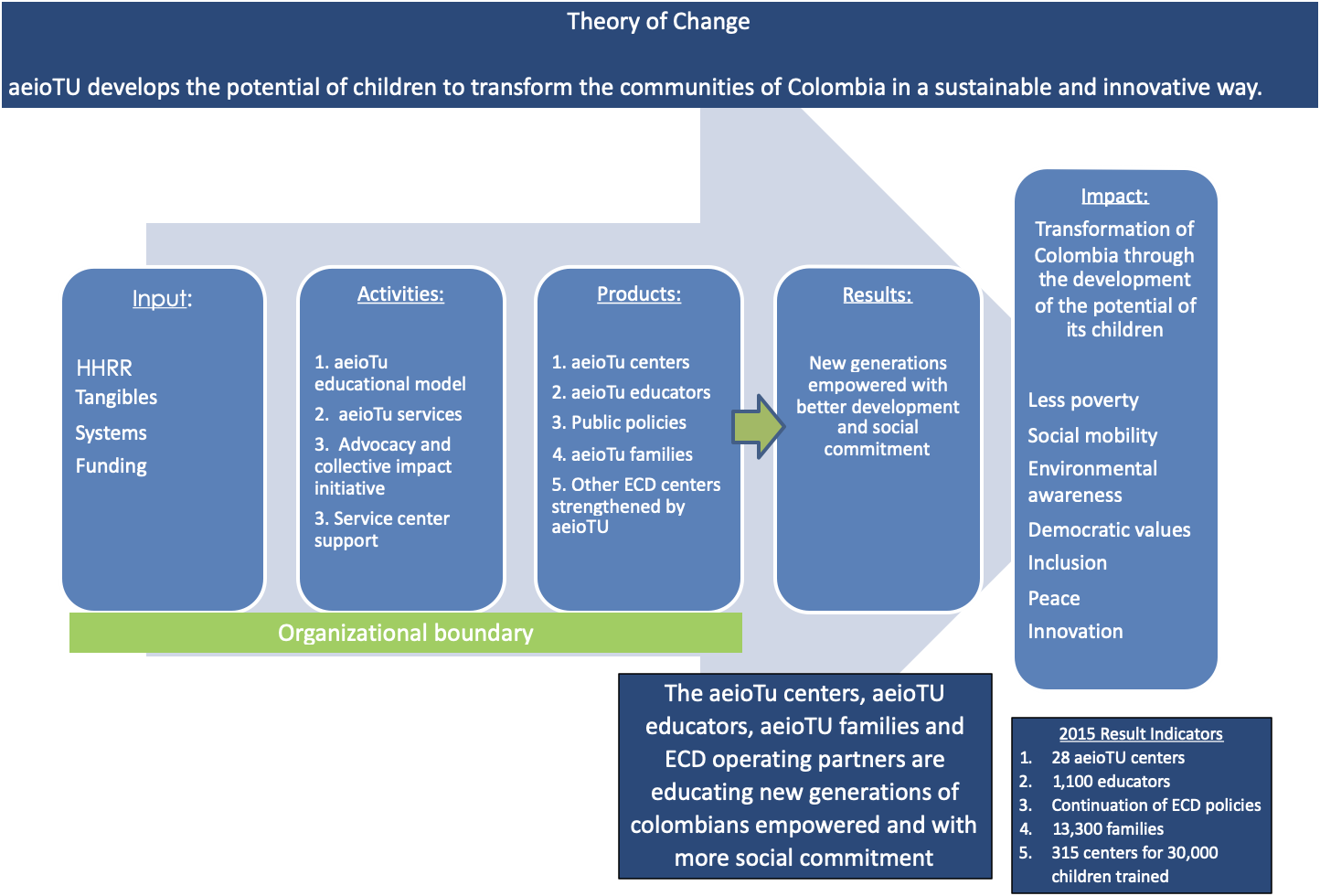

Supplement: Supplementary file 3 [file Image_3.png]
